# Serum pro-oxidant/antioxidant balance in term versus preterm neonates

**DOI:** 10.1097/MD.0000000000031381

**Published:** 2022-11-04

**Authors:** Hassan Boskabadi, Majid Ghayour-Mobarhan, Amin Saeidinia

**Affiliations:** a Pediatric Department, Faculty of Medicine, Mashhad University of Medical Sciences, Mashhad, Iran; b Biochemistry of Nutrition Research Center, School of Medicine, Mashhad University of Medical Sciences, Mashhad, Iran; c Booali Research Center, Pharmaceutical Sciences Division, Mashhad University of Medical Sciences, Mashhad, Iran.

**Keywords:** oxidative stress, preterm neonates, pro-oxidant antioxidant balance, term

## Abstract

The oxidant/antioxidant status balance is a process that begins before birth and premature infants are particularly susceptible to oxidative stress. According to the mechanisms of oxidative stress and lack of study in this field, in this prospective study, we aimed to compare the levels of serum pro-oxidant/antioxidant balance (PAB) in preterm versus term babies. This was a prospective cross-sectional study that was performed in Ghaem hospital, a university tertiary hospital, in Mashhad, Iran. The study population included all term and preterm neonates who were admitted to the hospital within birth time. In our study, 324 neonates were included. One hundred ninety-eight neonates were preterm (61.1%) and others were term (38.9%). There was a significant difference between PAB levels in term and preterm neonates. Serum PAB level was significantly lower in preterm neonates rather than in term neonates (21.86 ± 21.01 vs 50.33 ± 31.69; *P* = .001). There was also a significant negative correlation between PAB levels and gestational age. According to previous investigations, we showed for the first time in our study that PAB is lower in preterm newborns rather than in term ones.

## 1. Introduction

Free radicals are molecular species with an unpaired electron in the outer shell, which renders them highly reactive and unstable. FRs containing oxygen may be termed reactive oxygen species (ROS). The accumulation of reactive FRs, beyond the capacity of the endogenous antioxidant defense system to scavenge them, results in damage to DNA, proteins, and lipids that compromise cell function, leading to cell death via apoptosis or necrosis.^[1,2]^ Fetal life occurs in a relatively hypoxic environment. Hypoxia is necessary for the development and growth of the fetus. Under normal circumstances, the fetal-to-neonatal transition causes physiological oxidative stress (OS), which enhances the antioxidant defense and pulmonary surfactant maturation.^[3]^

The oxidant/antioxidant status balance is a process that begins before birth,^[4]^ and premature infants are particularly susceptible to OS.^[5,6]^ Most of the complications of prematurity, such as bronchopulmonary dysplasia, retinopathy of prematurity, necrotizing enterocolitis, intraventricular hemorrhage, periventricular leukomalacia, and punctate white matter lesions, appear related to OS,^[7,8]^ mostly occurring due to a mismatch between the free radical production and the anti-oxidative capacity of the premature neonate.^[6]^

Moreover, antioxidant defense mechanisms are incompletely developed or deficient in preterm newborns.^[9]^ Preterm infants show reduced antioxidant defense mechanisms, including decreased levels of vitamin E, β-carotene, melatonin, ceruloplasmin, transferrin, and erythrocyte superoxide dismutase (SOD).^[6]^ In a study on 100 preterms and 100 full-term neonates, plasma levels of vitamin A, vitamin E, and catalase were found significantly lower while plasma level of malondialdehyde (MDA), a marker of lipid peroxidation, was significantly higher in the preterm than in the full-term newborns, especially in those ones who developed necrotizing enterocolitis or bronchopulmonary dysplasia.^[10]^ Premature babies have both immature lungs and antioxidant defense systems and often require oxygen supplementation to overcome respiratory distress. The combination of hyperoxia, which enhances the generation of ROS, and low antioxidants cause OS, inflammation, and even apoptosis, thus increasing mortality and morbidity.^[11]^

According to the mechanisms of OS and lack of study in this field, in this prospective study, we aimed to compare the levels of serum pro-oxidant/antioxidant balance (PAB) in preterm versus term babies.

## 2. Methods

### 2.1. Study population

This was a prospective cross-sectional study that was performed in Ghaem hospital, a university tertiary hospital, in Mashhad, Iran between 2015 and 2019. The study population included all term and preterm neonates who were born in Ghaem hospital within birth time. This investigation was approved by the Ethical Committee of Mashhad University of Medical Sciences. We obtained parents’ informed written consent before entry into the study. The term low birth weight. ‘Low birth weight (LBW) has been defined as the first weight recorded within hours of the birth of fewer than 2500 g between 33 and 37 weeks gestational age. Very low birth weight is accepted as less than 1500 g at lower than 32 weeks gestational age.

### 2.2. Eligibility

We recruited all babies born in our center during the study interval without any abnormal examination, known anomalies in perinatal assessment, hypotonia, bradycardia, low Apgar score, requiring cardiopulmonary resuscitation, or need to use accessory oxygenation. We excluded neonates with severe birth defects, respiratory distress, positive history of eclampsia/preeclampsia in mothers, birth asphyxia, suspicious of sepsis, hemolytic hyperbilirubinemia, Rh or ABO incompatibility, and pathologic jaundice neonates.

### 2.3. Data collection

We completed a checklist for each neonate including gestational age, gender, birth weight, and Apgar score. Moreover, mothers’ data included age, parity, pregnancy-related problems, mode of delivery, and blood type were completed. Babies after sampling and data collection underwent routine care if necessary during their hospital admission. Blood samples were taken from the umbilical cord (1 cc for each neonate) and were used for PAB measurement after centrifuge and separation of serum. For this measurement, the separated blood samples were transported to Bu-Ali Research Institute.

### 2.4. PAB measurement

The solutions were prepared as indicated. The standard solutions were made by mixing varying proportions (0%–100%) of 250 μM hydrogen peroxide with 3 mM uric acid in 10 mM NaOH. In order to prepare the 3,3′,5,5′-tetramethylbenzidine (TMB) cation, 60 mg of TMP powder was dissolved and mixed well at a ratio of 10 to 20 mL of the solution. Then, the prepared solution was placed in the dark for 2 hours. Next, 25 units of peroxidase enzyme were added to 20 mL of the solution and placed at 20°C. For the preparation of the TMB solution, 200 mL of TMB was added to 10 mL of acetate buffer (0.05 M buffer, pH 5.8); the working solution was prepared by mixing 1 mL of TMB cation with 10 mL of TMB solution. The prepared solution was placed in a dark, dry place for 2 minutes. Next, 10 μL of each sample was mixed with 200 μL of the working solution and placed in a 96-well plate in the dark at 37°C for 12 minutes. At the end of the process, 100 μL of 2N Hall was added to each well and measured in an ELISA plate reader at 450 nm and 620 nm wavelengths. PAB values were calculated on the standard curve as previously published.^[12]^

### 2.5. Statistical analysis

Data were entered in SPSS-20 software (IBM, Chicago, IL). The continuous values were indicated as mean ± SD. An independent *t* test was used for continuous variables. Parametric and nonparametric correlations were assessed using Pearson correlation coefficients and Spearman correlation coefficients, respectively. *P* value < .05 was considered statistically significant.

## 3. Results

In our study, 324 neonates were included. One hundred ninety-eight neonates were preterm (61.1%) and others were term (38.9%). Table 1 shows the demographic data of neonates.

**Table 1:** Demographic data of neonates.

Variable	Term (n = 126) (n, %)	Preterm (198) (n, %)		*P* value
		Early (n = 101)	Late (n = 97)	
Gender	Male (76, 60.3)	Male (44, 43.5)	Male (51, 52.5)	.141
	Female (50, 41.67)	Female (57, 56.5)	Female (46,47.4)	
Type of delivery	NVD (48, 41.4)	NVD (28, 24.1)	40 (34.5)	.120
	CS (77, 37.4)	CS (72, 35)	CS (57, 27.7)	

CS = cesarean section, NVD = normal vaginal delivery.

The mean birth weight of participants was 3267.19 ± 446.35 g in term and 1658.78 ± 644.97 g in preterm neonates. Other perinatal factors are listed in Table 2.

**Table 2:** Differences between perinatal factors in term and preterm neonates.

Variable	Gestational age	Mean	Std. deviation	*P* value
Birth weight	Preterm (n = 198)	1658.78	644.97	.001^*^
	Term (n = 126)	3267.19	446.35	
Apgar score of first minute	Preterm (n = 198)	7.50	1.67	.001^*^
	Term (n = 126)	9.42	0.67	
Apgar score of fifth minutes	Preterm (n = 198)	8.91	1.31	0.001^*^
	Term (n = 126)	9.82	0.38	
Maternal age	Preterm (n = 198)	28.69	6.10	0.368^*^
	Term (n = 126)	27.92	4.53	
Parity	Preterm (n = 198)	2.12	1.27	0.002^*^
	Term (n = 126)	2.59	1.41	
PAB level	Preterm (n = 198)	21.86	21.01	0.001^†^
	Term (n = 126)	50.33	31.69	

PAB = pro-oxidant/antioxidant balance.

* Independent *t* test.

† Mann–Whitney test.

**Figure 1.**
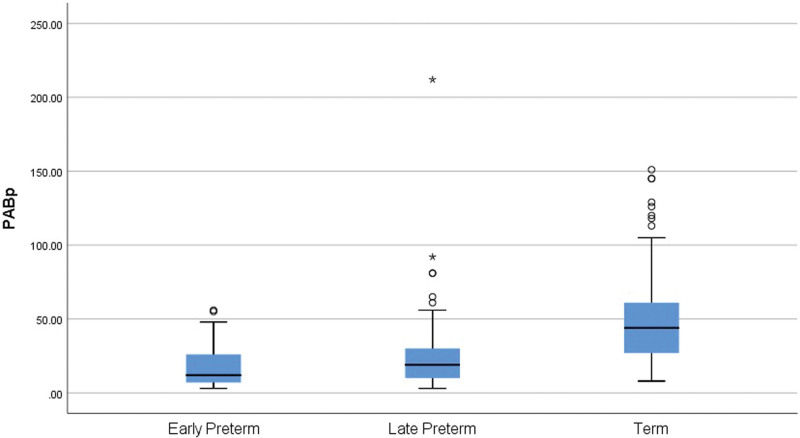
The difference between PAB plasma levels according to gestational age category. PAB = pro-oxidant/antioxidant balance.

As can be seen in Table 2, there was a significant difference between PAB levels in term and preterm neonates. Serum PAB level was significantly lower in preterm neonates rather than in term neonates (21.86 ± 21.01 vs 50.33 ± 31.69; *P* = .001). It is also shown in Figure 1 the difference between PABp levels according to gestational age categories.

**Figure 2.**
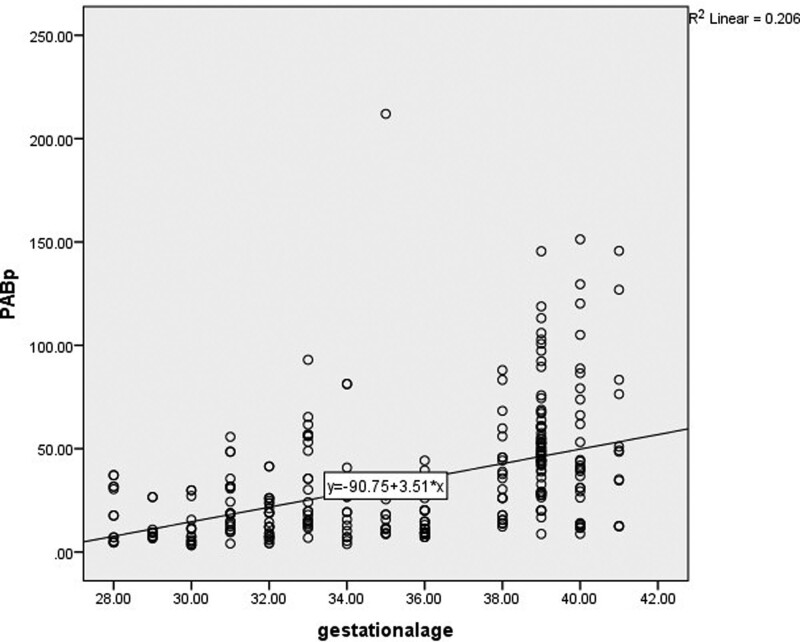
Correlation between PAB (pro-oxidant/antioxidant balance) level and gestational age.

There was also a significant negative correlation between PAB level and gestational age by number (*r* = –0.547, *P* < .001). Figure 2 shows the correlation between PAB level and gestational age.

Linear regression analysis showed that birth weight (*P* = .005) and gestational age (*P* = .002) were predictors of PAB level in our study population.

## 4. Discussion

To the best of our knowledge, this study was of limited investigation that compared the level of PAB level in term versus preterm neonates. Our findings showed that PAB level was significantly lower in preterm neonates. There are different studies on the neonatal populations about PAB levels. In a study, it was shown that there was no significant relationship between umbilical cord blood pro-oxidant antioxidant balance and type of delivery.^[13]^ In another study, it was shown that a pathological increase in bilirubin levels irrespective of its neurotoxic properties can change the PAB in favor of antioxidants.^[14]^ In asphyxia, it was demonstrated that PAB in combination with hypoxic-ischemic encephalopathy grade might have a better predictive value for the prognosis of asphyxiated babies and predicting future neurologic problems in asphyxiated term infants.^[15]^

There are more data on other oxidative indexes in preterm and term neonates. It was shown in previous studies that newborn preterm neonates had high levels of OS biomarkers. These markers were evaluated such as plasma, MDA-hemoglobin,^[16]^ F2-isoprostane,^[17]^ MDA,^[10,18,19]^ erythrocyte membrane hydroperoxide levels,^[20]^ and lipid peroxidation^[21]^ in preterm newborns clearly show higher levels of lipid peroxidation markers. It is also demonstrated that in comparison with full-term neonates, there are higher levels of 8-hydroxy-2-deoxyguanosine (8-OHdG),^[22]^ protein carbonyl^[18,23]^ and desferrioxamine chelate iron^[17]^ in preterm newborns. Compared to full-term birth, preterm newborns also enhance plasma nonprotein bound iron concentration,^[17,23]^ which can lead to oxidative damage through the Fenton reaction. Some studies reported that OS levels are negatively correlated with gestational age.^[17,18,22,24,25]^ In our study, this correlation was also confirmed. Our findings showed that preterm neonates had lower levels of PAB in comparison with term neonates. This strengthens the hypothesis that prematurity itself is at least partly capable of higher OS in preterm newborns.

This can be because of the lower levels of different antioxidant biomarkers. It is explored that at birth, SOD activity in both blood^[19,26]^ and erythrocytes,^[20,27]^ catalase activity in blood^[10]^ as well as cytosolic glutathione peroxidase in erythrocytes^[20]^ are lower in preterm than in full-term newborns. Preterm newborns also exhibit lower levels of nonenzymatic antioxidants such as erythrocyte vitamins.^[28]^ Many physiological mechanisms can induce the higher OS levels observed in preterm newborns. It is known that OS levels in mothers vary during pregnancy^[29]^ and this can be because of increased OS in the placenta.^[29,30]^ This OS in the placenta is probably necessary for its development by regulating the proliferation, differentiation, and invasion of trophoblasts, promoting placental angiogenesis, and regulating autophagy and apoptosis required for placentation.^[31]^ However, a high level of systemic OS in mother can be related to preterm labor and prematurity.^[32]^ Therefore, higher OS level in pregnant women might lead to dysfunction of placenta or other damages that lead to preterm labor and be responsible for the high OS level observed in preterm newborns by direct blood exchange in the placenta.^[28]^ On the other hand, preterm newborns have an immature antioxidant system, as the last weeks of pregnancy correspond to the maturation and upregulation of the fetus’s antioxidant system^[10]^ and the transfer of some antioxidants from the mother to the fetus.^[19]^ This may explain why babies born early in their third-trimester exhibit lower concentrations of antioxidants. Moreover, preterm newborns might need several medical interventions because of their immature state and this can also induce ROS generation.

Although in previous studies, there were many biomarkers of OS evaluated in preterm versus term newborns, there is still a need to find the applicable tests to find this hypothesis. In this study, we used an approach with potential clinical applications in oxidant/antioxidant assays, which was the PAB technique. The assay is a new strategy to determine the pro-oxidant load and antioxidant capacity in a single assay. The assay provides a general view of the oxidant/antioxidant status of the patients in one single experiment.^[33]^ Despite the novelty and relatively large sample size, there were some limitations in this study. Measurement of other antioxidant biomarkers like SOD, catalase, MDA, and glutathione peroxidase and finding the correlation between them and PAB could reinforce the results of this hypothesis. Lack of long-term follow-up of neonates’ outcomes and no other biochemical and maternal factors for finding the predictive value of PAB, were other limitations of this investigation.

## 5. Conclusion

According to previous investigations, we showed for the first time in our study that PAB is lower in preterm newborns rather than in term ones. This is in line with the hypothesis that OS is higher in preterm neonates. Targeting OS in preterm neonates as an applicable clinical index can complete the pathway of effects of OS in preterm newborns. More studies targeting PAB are needed in the future to prevent the poor prognosis of preterm newborns with chronic disorders in childhood.

## Acknowledgments

We thank the deputy of research at Mashhad University of Medical Sciences and healthcare workers of the neonatal intensive care unit especially Mrs. Eskandari, Mrs. Ramezani, Mrs. Nikooseresht, Mrs. Aelam, Mrs. Hakimi, and laboratory colleagues especially Mrs. Tavallayi and Mr. Asaran. We also thank Mr. Mehrabi and Mr. Parvizi for their cooperation.

## Author contributions

**Conceptualization:** Hasan Boskabadi, Majid Ghayour-Mobarhan.

**Data curation:** Hasan Boskabadi.

**Investigation:** Hasan Boskabadi, Majid Ghayour-Mobarhan.

**Methodology:** Hasan Boskabadi, Majid Ghayour-Mobarhan.

**Writing – original draft:** Hasan Boskabadi, Majid Ghayour-Mobarhan, Amin Saeidinia.

**Writing – review and editing:** Hasan Boskabadi, Majid Ghayour-Mobarhan, Amin Saeidinia.
